# Whole Slide Imaging (WSI) in Pathology: Emerging Trends and Future Applications in Clinical Diagnostics, Medical Education, and Pathology 

**DOI:** 10.30699/ijp.2025.2044210.3367

**Published:** 2025-07-01

**Authors:** Sadegh Masjoodi, Mohammad Hossein Anbardar, Mansoureh Shokripour, Navid Omidifar

**Affiliations:** 1 Shiraz Neuroscience Research Center, Shiraz University of Medical Sciences, Shiraz, Iran; 2 Department of Pathology, School of Medicine, Shiraz University of Medical Sciences, Shiraz, Iran

**Keywords:** Telepathology, Clinical Pathology, Artificial Intelligence, Deep Learning, Computing Methodologies

## Abstract

**Background & Objective:**

Whole Slide Imaging (WSI) has emerged as a transformative technology in the fields of clinical diagnostics, medical education, and pathology research. By digitizing entire glass slides into high-resolution images, WSI enables advanced remote collaboration, the integration of artificial intelligence (AI) into diagnostic workflows, and facilitates large-scale data sharing for multi-center research.

**Methods:**

This paper explores the growing applications of WSI, focusing on its impact on diagnostics through telepathology, AI-powered diagnoses and precision medicine, and educational advancements. In this report, we will highlight the profound impact of WSI and address the challenges that must be overcome to enable its broader adoption.

**Results & Conclusion:**

Despite its many advantages, challenges such as infrastructure limitations and regulatory issues need to be addressed for broader adoption. The future of WSI lies in its ability to integrate with cloud-based platforms and big data analytics, continuing to drive the digital transformation of pathology.

## Introduction

One of the recent advancements in pathology is **Whole Slide Imaging (WSI)**, a technique that involves scanning glass slides to produce high-resolution digital images that can be viewed and manipulated like any other digital image (1). Functionally analogous to traditional light microscopy—but with greater ease of use, enhanced interactivity, and remote accessibility—WSI is transforming clinical diagnostics, medical education, and pathology research. The shift from conventional microscopy to WSI offers numerous advantages, including remote collaboration, streamlined and standardized diagnostic workflows, and the integration of artificial intelligence (AI) into clinical practice (2–4).This report aims to examine the impact of WSI on clinical diagnostics, education, and research, with a focus on key areas of interest and potential future applications. It also discusses the significant influence of WSI on modern pathology and highlights the challenges that must be addressed to support its broader implementation.

## Materials and methods

### WSI in Clinical Diagnostics

WSI is enhancing clinical diagnostics by offering digital and intelligent tools that improve diagnostic accuracy, increase efficiency, and simplify collaboration. Owing to its applications in telepathology, AI integration, and the move toward standardized diagnostic procedures, WSI is emerging as a transformative technology in modern medical diagnostics (5,6).

#### A. Enhancing Diagnostic Accuracy

WSI enables pathologists to examine digital slides—virtual representations of tissue samples—on computer screens. These high-resolution images allow precise manipulation, including zooming, panning, and rotating, which provides a clearer and more detailed view than traditional light microscopy. This enhanced visual capacity allows for the detection of subtle abnormalities that might otherwise be overlooked (7).

This capability is particularly valuable in the diagnosis of complex diseases, such as cancer, where accurate identification of abnormal tissue architecture significantly influences differential diagnosis, treatment planning, and follow-up assessments (8). For instance, in cancer diagnosis, WSI facilitates more accurate assessment of cancer cell morphology due to its superior image clarity (9).

Small tissue biopsies—such as skin samples or needle-core biopsies—often require meticulous scrutiny. Digital slides allow pathologists to zoom into specific areas of interest, supporting a more detailed and focused analysis (10,11). Additionally, WSI platforms support the simultaneous review of multiple slides, which enables comparative evaluations and further enhances diagnostic confidence (12).

Additionally, WSI enhances data storage and retrieval, enabling easier tracking of patient histories and revisiting prior diagnoses for follow-up care. Unlike glass slides, which may degrade over time, digital WSI images are preserved without the risk of physical damage or fading, ensuring long-term accessibility and integrity of diagnostic materials (13,14).

**Fig. 1:**
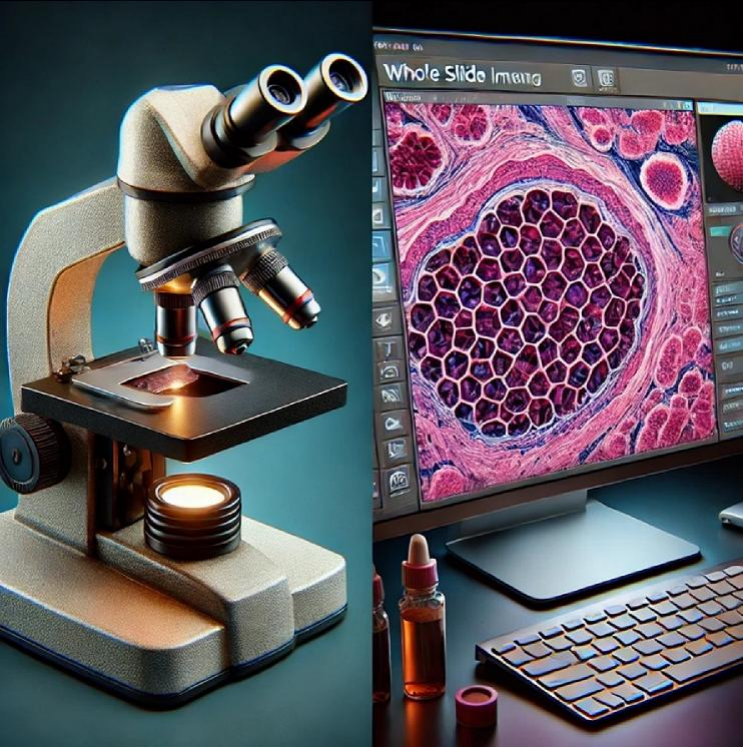
Traditional Microscopy vs Whole Slide Imaging

#### B. Telepathology and Remote Diagnostics

Telepathology—one of the most impactful features enabled by WSI—facilitates the remote viewing, sharing, and diagnosis of digital pathology slides. This capability is particularly valuable in healthcare systems where expert pathologists are scarce or not physically accessible. By digitizing glass slides and uploading them to secure digital platforms, WSI enables pathologists across different geographic locations to review, consult, and collaborate on cases in real time. Such remote access enhances diagnostic accuracy and ensures timely care, regardless of a patient's location (15–17).

Intraoperative consultations, such as those involving frozen section pathology, particularly benefit from telepathology. WSI enables the rapid digitization and remote review of frozen tissue samples, allowing pathologists to provide real-time feedback to surgeons. This is especially beneficial in urgent or resource-limited settings, where immediate access to a pathologist may not be feasible.

Telepathology proved to be indispensable during the COVID-19 pandemic, when physical access to hospitals and laboratories was severely restricted (18,19). In such circumstances, pathologists were able to review virtual slides from home or remote offices, ensuring continuity in diagnostic workflows with minimal disruption (20). Furthermore, WSI allows pathologists to participate in multidisciplinary team meetings, such as tumor boards, where collaborative input is essential for accurate diagnosis and optimal treatment planning (21,22).

WSI also expands access to pathology services in underserved or remote regions, where specialized professionals may not be available (23). By transmitting digital slides over secure networks to specialized centers, patients in rural or low-resource areas can receive expert diagnostic input. Additionally, WSI enables second-opinion consultations for rare or complex cases without the logistical barriers associated with physical slide transport, thereby overcoming a significant limitation in traditional pathology workflows (24).

#### C. Integration of AI in Diagnostics

The integration of artificial intelligence (AI) into WSI platforms is one of the most significant developments in digital pathology. AI-powered image analysis tools augment the diagnostic capabilities of pathologists by assisting in the detection, classification, and quantification of abnormalities in digital slides (25). Through machine learning algorithms trained on large, annotated datasets, AI can recognize morphological patterns—such as cancer cell features—and highlight them for rapid decision-making by the pathologist (26,27).

Advanced AI models, such as deep learning neural networks, are capable of performing complex pattern recognition and quantitative analysis. These tools can detect minute histological changes that might be overlooked by the human eye. For example, in cancer diagnostics, AI can analyze digital slides and flag suspicious areas for closer review, thereby improving diagnostic accuracy and consistency (28,29). The combination of AI and WSI not only enhances accuracy and speed but also streamlines workflows, significantly reducing the time required for case evaluation and diagnosis.

Moreover, AI tools are particularly useful for high-volume, routine screening tasks, allowing pathologists to allocate their time to more complex and nuanced cases. In breast cancer screening, for instance, AI algorithms can efficiently process large numbers of biopsy slides, flagging only those that require further human review. This improves turnaround times and optimizes resource utilization (8,19,30).

Integrated AI–WSI platforms also support the advancement of personalized medicine. Efficient data archiving enables retrospective analysis, while AI algorithms facilitate the identification of specific biomarkers and genetic mutations in tumor tissues—information that can inform targeted therapeutic strategies (31). As deep learning–based classifiers continue to evolve, AI is poised to become an indispensable tool in digital pathology, offering robust support for clinical decision-making.

### WSI in Medical Education

Whole Slide Imaging (WSI) has the potential to fundamentally transform medical education, particularly in the teaching of histology and pathology. As noted by Pantanowitz et al, WSI can effectively replace traditional light microscopy in many educational settings, offering enhanced flexibility, accessibility, and interactivity in the learning process (32). This shift allows students to engage with high-quality virtual slides from any location and at any time, thereby revolutionizing the way pathology is taught and learned.

#### A. Virtual Microscopy and Accessibility

By enabling students to interact with digital slides, WSI significantly improves the accessibility of pathology education. Unlike traditional glass slides, which require physical presence in a laboratory and are often limited to viewing through multi-head microscopes by only a few users at a time, digital slides can be accessed on any internet-connected device. There are no restrictions on the number of users who can simultaneously view the same slide, which greatly enhances collaborative learning.

This digital accessibility is especially valuable for students who wish to review material outside of scheduled class times or for those who are unable to attend in-person laboratory sessions due to geographic, logistical, or health-related constraints (33–35). Furthermore, WSI supports asynchronous learning and remote instruction, making it a powerful tool for modern medical curricula, including distance education programs. 

Furthermore, WSI offers students access to a wide range of pathology cases that may not be readily available in a typical teaching environment or laboratory. Rare or unique cases can be digitized and shared, providing learners with the opportunity to study a broader variety of specimens (36). This diversity enriches the educational experience and better prepares students for the range of diseases they are likely to encounter in clinical practice. At several institutions—such as the University of Pittsburgh Medical Center—WSI has been integrated into graduate medical education programs, enabling students to explore comprehensive digital slide repositories spanning multiple pathology subspecialties (37). These curated teaching sets allow students to learn at their own pace, fostering a deeper understanding of pathological processes.

**Fig. 2:**
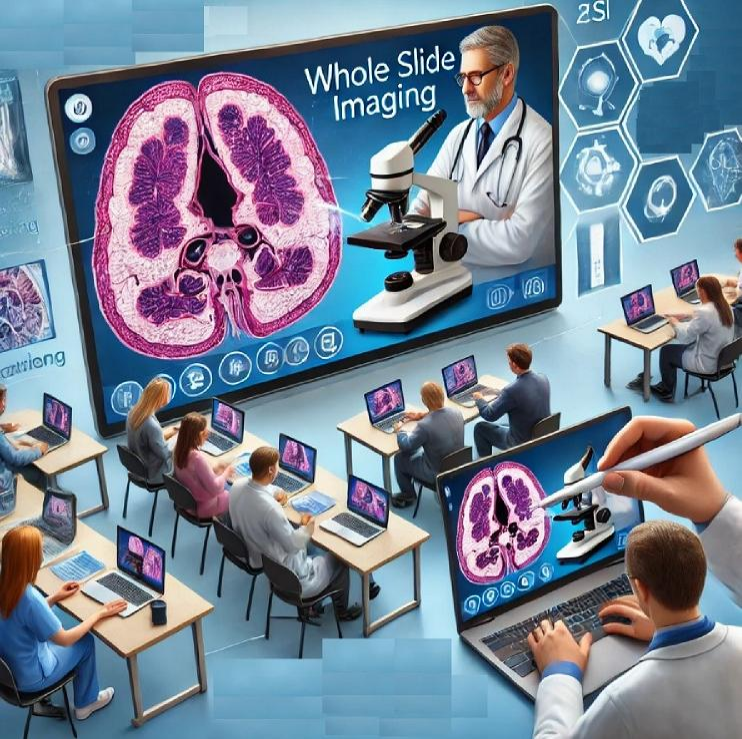
WSI in Medical Education: An infographic or diagram showing how WSI supports different educational elements like virtual microscopy, interactive learning, and remote collaboration. This could include examples like institutions adopting WSI and the benefits for pathology training.

#### B. Interactive and Collaborative Learning

WSI promotes interactive and collaborative learning by allowing students and instructors to engage with digital slides in real time or asynchronously. Through standard WSI platforms, students can zoom, annotate, and highlight areas of interest, replicating many functions of a traditional microscope with enhanced usability. This interactivity encourages active engagement and deeper learning, often leading to a better grasp of complex concepts compared to traditional passive methods (38).

Instructors can assess student understanding through shared annotations and comments, offering individualized feedback and guiding students through the diagnostic process in greater detail. WSI also supports collaborative environments in which students analyze slides together, share interpretations, and discuss findings with peers. Group study and peer-to-peer feedback are further enabled by remote access to digital slides, making collaboration feasible regardless of physical location (24,39).

WSI also enhances teaching in laboratory settings. Traditional glass slide collections are often limited in number, restricting access to critical specimens. In contrast, digital slides can be simultaneously accessed by multiple students, ensuring equal exposure to key educational materials—particularly important during practical exams and assessments, when all students must evaluate the same slide concurrently (40). Another valuable feature of WSI is the ability to save and share annotated slides. Faculty can prepare curated teaching sets that emphasize diagnostic features, providing students with high-quality resources for self-study (41). Likewise, students can share their annotations with peers, fostering collaboration and discussion that deepen understanding.

#### C. Pathology Training and Competency Assessments

WSI has also transformed pathology residency training. Digital teaching sets allow programs to offer standardized collections that include both common and rare cases, ensuring equitable access to high-quality diagnostic materials across trainees. These repositories overcome the limitations of traditional glass slide collections, which are often difficult to maintain and distribute consistently.

Beyond education, WSI platforms also support competency-based assessments. Many systems incorporate tracking tools that monitor user interactions, such as time spent on specific slide regions, zooming patterns, and diagnostic pathways. These metrics provide valuable insights into residents' diagnostic strategies and can help identify areas where additional instruction is needed.

WSI-based assessments are gaining adoption in board certification processes. For instance, the American Board of Pathology has begun incorporating digital pathology into certification examinations, reinforcing the need for pathologists to demonstrate proficiency with WSI technologies. This trend underscores the increasing role of digital tools in clinical practice and highlights the necessity of formal training in digital pathology for current and future pathologists.

### WSI in Pathology Research

The adoption of WSI has created significant opportunities for advancement in pathology research, particularly in data-intensive and multicenter collaborations. Digitizing histological slides allows researchers to share data efficiently, collaborate across institutions, and perform large-scale image analyses that were previously impractical or impossible with physical slides (12,42).

#### A. Big Data and Image Analysis

The digitization of pathology slides has enabled the generation and analysis of vast pathological datasets—often referred to as “big data.” The use of WSI in research supports the acquisition of large volumes of image data, which can be applied to the study of disease patterns, tissue morphology, and diagnostic accuracy. This is particularly valuable in cancer research, where large-scale studies are essential for identifying trends and correlations across thousands of cases (28).

AI- and deep learning–based image analysis tools have been developed to process these datasets, allowing researchers to detect patterns and anomalies that would be difficult or impossible to identify manually (43). For instance, in oncologic research, AI algorithms can analyze digital slides to quantify tumor size, cellular morphology, and biomarker expression, thereby providing insights into disease progression and therapeutic response (25).

Moreover, the ability to store and share digital slides facilitates collaborative, multi-institutional studies by allowing data from multiple centers to be pooled and analyzed collectively. Such collaborative efforts enhance the statistical power of studies and increase the reliability and reproducibility of research findings (44).

#### B. Data Sharing and Collaboration

WSI significantly enhances collaboration among researchers by enabling digital slide sharing via secure online platforms. In the past, physical slides had to be shipped between institutions for joint studies, a process that was both time-consuming and fraught with risk of damage (15). WSI eliminates these logistical barriers, making collaboration faster, safer, and more efficient.

This advancement has accelerated progress in fields such as oncology, where multi-center research is commonplace. Additionally, WSI has facilitated the emergence of virtual workshops and global conferences, where researchers can present their findings and receive real-time feedback using digital slides. The ability to discuss complex cases across institutions enables rapid problem-solving and opens new research avenues across disciplines.

### Tracking, Tutoring, and E-Learning

WSI platforms also support educational innovations through interaction tracking, personalized tutoring, and e-learning analytics. These features allow educators to assess users’ diagnostic behavior and improve training strategies based on objective data.

#### A. Slide Navigation Tracking

Many WSI systems now incorporate tools that track how users navigate digital slides. These tools collect data on where users zoom, how long they dwell on specific areas, and how they move across a slide (32,46–48). This tracking is particularly valuable in educational settings, as it provides quantitative insights into students’ and trainees’ diagnostic approaches.

Educators can use these data to evaluate learner performance and offer targeted feedback. Research has shown that expert pathologists and trainees exhibit different navigation behaviors: for example, experienced pathologists tend to spend more time on diagnostically relevant regions and employ more precise zooming behavior. Understanding these differences helps educators refine their instructional methods to bridge skill gaps and improve learning outcomes (49,50).

#### B. SlideTutor

*SlideTutor* is a notable example of an educational platform that leverages WSI’s tracking capabilities to provide real-time, adaptive feedback for learners (51). This tool monitors how students interact with digital slides and offers guidance when diagnostic errors are made. Operating under a "virtual apprenticeship" model, *SlideTutor* has been shown to significantly enhance diagnostic accuracy and reporting skills after just a few hours of use.

By encouraging students to focus on diagnostically important areas and offering individualized feedback, *SlideTutor* supports the development of structured slide review strategies. Its adaptability allows it to tailor instruction to each student’s needs, making it an effective platform for personalized learning and accelerated competency development.

### Tele-Education and Virtual Tumor Boards (Teleconferencing)

WSI could become an essential tool in tele-education and particularly in the context of virtual tumor boards, where clinicians and pathologists collaborate to discuss complex cases (52-54). WSI could facilitate remote case presentations during teleconferences, where clinicians and pathologists review digital slides in real-time (55). During these virtual meetings, participants can zoom in on specific areas of a slide, annotate findings, and ask questions, just as they would in a traditional tumor board setting. This real-time collaboration allows for more dynamic discussions and ensures that all members of the multi-disciplinary team are on the same page. The use of WSI in tumor boards has been shown to enhance the educational value of these meetings, as it allows clinicians to engage more actively with the pathology findings (21). Pathologists can prepare slides more efficiently for tumor board presentations, as WSI eliminates the need for time-consuming slide photography (56, 57). 

### Proficiency Testing and Medical Examinations

WSI is increasingly being used in proficiency testing and medical examinations, where digital slides are used to assess diagnostic skills.

#### A. Proficiency Testing 

Institutions such as the College of American Pathologists (CAP) have implemented WSI for proficiency testing, allowing participants to review digital slides and make diagnostic decisions online. This ensures that all participants are evaluated using the same high-quality slides, standardizing the testing process and removing the variability associated with physical slide handling. WSI is particularly useful for performance improvement programs, where pathologists review digital slides to assess their diagnostic accuracy. These programs are essential for maintaining high standards of care, and WSI ensures that participants are evaluated using consistent, reproducible materials (58).

#### B. Medical Examinations

WSI is now being used in medical exams, particularly in practical exams where students are required to analyze digital slides. The WebMicroscope platform, for example, has been successfully used at Poznan University of Medical Sciences in Poland to administer pathology exams to dental students (59). This platform allows students to interact with digital slides during their exams, mimicking the real-world diagnostic process.

The use of WSI in exams offers several advantages over traditional methods. Students can access the same slides simultaneously, ensuring that all participants are evaluated on the same material. In addition, WSI allows for more complex, interactive questions, where students must navigate through a slide to identify specific diagnostic features. This approach better reflects the real-world challenges of diagnostic pathology and prepares students for clinical practice (38).

### Challenges and Future Trends

While WSI offers numerous advantages, several challenges must be addressed to enable its broader adoption. These include technical barriers related to file sizes and infrastructure, as well as regulatory issues surrounding its use in clinical diagnostics.

#### A. Overcoming Technological Barriers

WSI files are significantly larger than typical medical images, requiring considerable storage capacity and high-speed networks to support smooth viewing. Institutions must invest in robust infrastructure to accommodate these demands, which can be a challenge in low-resource settings. Viewing performance can also be affected by file size, with larger images necessitating more powerful computing systems and faster internet connections to ensure seamless access (4,60).

To address these challenges, many institutions have adopted cloud-based WSI platforms that allow for remote storage and access to digital slides. These systems offer scalable storage solutions, enabling large volumes of data to be managed without the need for extensive on-site infrastructure. However, the use of cloud-based WSI platforms raises important considerations related to data security and patient privacy. Ensuring compliance with healthcare regulations is essential to protect sensitive patient information (61–63).

#### B. Regulatory and Standardization Issues

As WSI becomes more deeply integrated into clinical workflows, it is critical to establish robust regulatory frameworks that ensure its safe and effective use—especially when AI-assisted diagnostics are involved. Regulatory agencies, such as the U.S. Food and Drug Administration (FDA), have begun developing guidelines for the approval and clinical use of digital pathology systems (64,65). Nonetheless, further efforts are needed to create standardized protocols for the use of WSI across diverse healthcare settings.

Standardization is essential for the broader adoption of WSI. Pathology departments must ensure interoperability between WSI systems and other healthcare technologies, including laboratory information systems (LIS) and electronic health records (EHRs). Seamless integration is necessary to streamline diagnostic workflows and support the routine incorporation of WSI into clinical practice (66,67).

## Conclusion

The Whole Slide Imaging (WSI) has revolutionized the fields of clinical diagnostics, medical education, and pathology research by enhancing accessibility, flexibility, and diagnostic accuracy. In clinical settings, WSI facilitates telepathology and integrates artificial intelligence to improve diagnostic precision. In medical education, it is replacing traditional microscopy in many institutions, offering interactive learning opportunities and enabling competency-based assessments. In research, WSI supports large-scale collaboration, data sharing, and advanced image analysis.

Despite these advances, challenges remain—including those related to infrastructure limitations, large file sizes, and regulatory oversight. The adoption of cloud-based platforms and continued development of AI-driven diagnostics present promising avenues for overcoming these barriers. As WSI technology continues to evolve, its influence will expand across healthcare domains, further propelling the digital transformation of pathology.
